# Dynamic in vitro 3D culture of cryopreserved human ovarian tissue: transcriptomic analysis by RNA sequencing

**DOI:** 10.1186/s13048-025-01896-9

**Published:** 2026-01-16

**Authors:** Qingduo Kong, Daniel Stavrev, Gohar Rahimi, Plamen Todorov, Cheng Pei, Evgenia Isachenko, Mahmoud Salama, Christine Skala, Volodimir Isachenko

**Affiliations:** 1 Department of Obstetrics and Gynecology, Medical Faculty, Research Group for Reproductive Medicine, Cologne University, Cologne, 50931 Germany; 2 Hospital of Obstetrics and Gynecology “Selena”, Plovdiv, 4002 Bulgaria; 3 Institute of Biology and Immunology of Reproduction of Bulgarian Academy of Sciences (BAS), Sofia, 1113 Bulgaria; 4 Medizinisches Versorgungszentrum AMEDES für IVF- und Pränatalmedizin in Köln GmbH, Cologne, 50968 Germany; 5 Department of Obstetrics, Gynecology and Reproductive Biology, College of Human Medicine, Michigan State University, Michigan, USA

**Keywords:** Human ovarian tissue, In vitro culture, TISSEEL fibrin, Cryopreservation, Thawing, RNA sequencing

## Abstract

**Purpose:**

To explore the in vitro 3D culture of ovarian tissue thawed in two different ways with TISSEEL Fibrin and assessed transcriptome differences by RNA sequencing.

**Methods:**

Human ovarian tissue samples were collected, and the fragments used for the experiments were obtained from tumor patients involved in a fertility treatment program. After cryopreservation (frozen and thawed after initial cryopreservation), with or without 3D culture with TISSEEL Fibrin. The culture flask is agitated at 75 times per minute using a rotary shaker during the entire culture process. Four experimental groups were formed. Frozen tissue after quick thawing at 100 °C (Group 1), frozen tissue after quick thawing at 100 °C and in vitro 3D culture for 7 days with TISSEEL Fibrin (Group 2), frozen tissue after slow thawing at 37 °C (Group 3), frozen tissue after slow thawing at 37 °C and in vitro 3D culture for 7 days with TISSEEL Fibrin (Group 4). All groups were followed by RNA sequencing and histological evaluation.

**Results:**

Hematoxylin-eosin (HE) staining has shown that the follicle cells have the tendency to develop and actively migrate into the fiber. Kyoto Encyclopedia of Genes and Genomes (KEGG) analysis shows that in comparison to groups 1 and 3 (thawing ovarian tissue without culture), Differential Expression Genes (DEGs) in groups 2 and 4 (thawing ovarian tissue with in vitro 3D culture), are mainly enriched and up-related in the lysosome pathway and protein processing in the endoplasmic reticulum pathway and mainly down-related in the cell adhesion molecules pathway.

**Conclusions:**

The technology of the described dynamic in vitro 3D-cultivation of ovarian tissue for 7 days with TISSEEL Fibrin is informative and demonstrates that it may promote follicular growth, actively migrate into the fiber, weaken cell adhesion, and cause little cell damage.

## Introduction

Ovarian tissue cryopreservation has emerged as a clinically feasible fertility preservation strategy, particularly for cancer patients, women with premature ovarian failure, and women with diminished ovarian function [1, 2]. It is a critical option for emergency situations and prepubertal patients who are not suitable for oocyte or embryo cryopreservation [3, 4]. Despite advances in cryopreservation and thawing methods, ovarian tissue still presents varying degrees of damage after cryopreservation (freezing and thawing), including damage to follicles, blood vessels, and stromal cells, which may compromise its functional integrity. In vitro culture of ovarian tissue after cryopreservation has shown the potential to enhance follicular viability and promote ovarian tissue recovery, highlighting its importance in optimizing fertility outcomes [5, 6].

In vitro culture of ovarian tissue includes various methods, direct culture, three-dimensional (3D) suspension culture, microfluidic culture, and 3D scaffold-based culture [7]. The direct culture method presupposes a placement of ovarian tissue fragments in a culture dish with medium. However, the lack of a 3D structural environment limits follicular development. In contrast, 3D suspension culture preserves tissue architecture and enhances follicular survival and maturation by preventing adhesion[8]. At the same time, structural instability of cells remains a challenge. Microfluidic culture, using microfluidic chips for precise control of nutrient supply, is well-suited for long-term culture, but the limitation is its technical complexity and high cost. The 3D scaffold culture method presupposes a placement of ovarian tissue into natural or synthetic biomaterials, facilitating cell-cell interactions, improving follicular viability, and advancing artificial ovary development. The selection of appropriate biomaterials is crucial, as they significantly influence tissue functionality and developmental outcomes [9–11].

3D scaffolds provide a structural framework that mimics the extracellular matrix, supporting cell attachment, proliferation, differentiation, and tissue formation. Various scaffold materials, including natural, synthetic, and composite biomaterials, each have different advantages and limitations [12–14].

Among them, the composite material TISSEEL Fibrin has shown good potential. TISSEEL is a fibrin sealant composed of two key components: a fibrinogen solution and a thrombin solution. When these two components are mixed during application, thrombin converts fibrinogen into fibrin, forming a stable, elastic clot that mimics the final stage of the natural blood coagulation process. This clot acts as a biological adhesive to promote hemostasis and tissue sealing. TISSEEL is biocompatible because it is derived from human plasma proteins that are carefully purified and treated to minimize the risk of viral transmission or immune reactions, allowing it to integrate safely with the tissues. TISSEEL Fibrin has excellent biocompatibility, provides structural support, and gradually degrades, making it a viable option for 3D scaffolds [15, 16].

In clinical practice, TISSEEL is widely used in surgical procedures to control bleeding, seal tissue, and promote wound healing, especially in cardiovascular, hepatic, and orthopedic surgeries where traditional sutures or staples may be ineffective or difficult to perform[16–18]. Therefore, based on the adhesive properties of TISSEEL fibrin, it can serve as an alternative to suture fixation in ovarian transplantation. By minimizing tissue compression and mechanical stress, it is expected to reduce transplant-related damage and promote the integration and development of ovarian tissue.

In tissue culture, TISSEEL’s application is primarily due to its excellent biocompatibility and tissue scaffold function. As a human fibrin-based bioglue, TISSEEL mimics the natural extracellular matrix (ECM) environment in vivo, providing a soft, moist, and biodegradable three-dimensional support structure for tissue es, helping to maintain cell viability and morphology. Its fibrin clots promote cell adhesion, proliferation, and differentiation, while allowing the free diffusion of nutrients and metabolites, thus supporting the long-term in vitro culture.

In previous studies, 3D scaffold culture based on TISSEEL Fibrin successfully supported ovarian cells’ development. In about seven days, primordial follicles were activated and developed into secondary follicles, and the artificial ovarian structure showed signs of degradation over time, demonstrating its good properties [19]. In addition to in vitro culture applications, minimizing mechanical damage during ovarian transplantation is critical to optimizing ovarian tissue viability and function [20].

In this study, transcriptomic analysis was used to comprehensively evaluate the advantages and limitations of TISSEEL-based 3D scaffold culture with the goal of optimizing the method for in vitro culture of ovarian tissue.

## Materials and methods

### Collection of samples and cryopreservation (freezing and thawing)

This study was conducted following the Declaration of Helsinki and approved by the Institutional Ethics Committee of Cologne University (applications 999,184 and 13–147) and by the Bulgarian Ethics Committee. Informed consent was obtained from patients whose ovarian tissue was collected for this study. Unless specified otherwise, all chemicals were obtained from Sigma Chemical Co. (St. Louis, MO, USA).

Cryopreservation (freezing and thawing) of ovarian tissue was performed according to our previously published protocol [21–25]. On the day of freezing, ovarian tissue fragments were placed in 20 mL of freezing medium at room temperature, which consisted of basal medium supplemented with 6% dimethyl sulfoxide (DMSO), 6% ethylene glycol, and 0.15 M sucrose. Tissue fragments were then transferred to standard 5 mL cryovials (Thermo Fisher Scientific, Rochester, NY, USA), prefilled with freezing medium, and frozen using an IceCube 14 S freezer (SyLab, Neupurkersdorf, Austria). The cryopreservation protocol was as follows: (1) starting temperature was − 8 °C; (2) samples were cooled from − 8 °C to − 34 °C at a rate of 0.3 °C/min; and (3) at − 34 °C, the cryovials were placed in liquid nitrogen. The freezing protocol also included an automated seeding step at − 8 °C.

#### Quick thawing

Tissue thawing was achieved by placing the cryovials at room temperature for 30 s, then immersing them in a 100 °C (boiling water) water bath for 60 s and draining the contents of the tubes into a solution to remove the cryoprotectant. The exposure time in boiling water was visually controlled by the presence of ice in the culture medium; once the ice reached a size of 2 to 1 mm, the tubes were removed from the boiling water, at which point the final temperature of the culture medium was between 4 and 10 °C. Within 5 to 10 s of thawing, the tissue fragments in the cryovials were drained into 10 mL of thawing solution (basal medium containing 0.5 M sucrose) in a 100 mL specimen container (Sarstedt, Neumbrecht, Germany). After 15 min of exposure of the tissue to sucrose, the cells were rehydrated stepwise as previously reported [21–25].

#### Slow thawing

This thawing method is the same as the quick thawing method above, except that the tissue is thawed by immersing the cryovial in a 37 °C water bath for 3 min until the ice has completely melted.

### Design of experiments

**Fig. 1:**
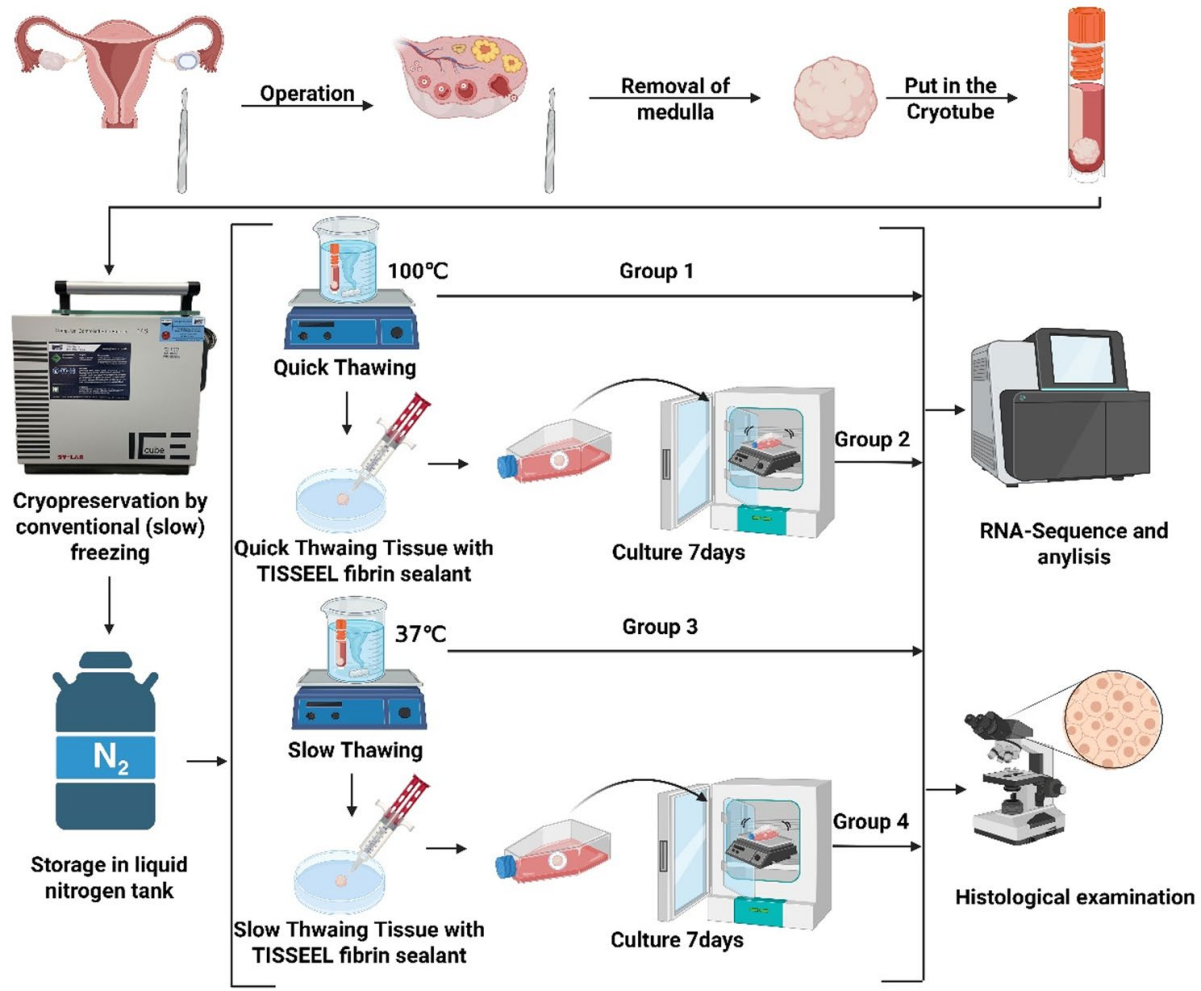
Design of experiments

Twelve ovarian tissue fragments were collected from four oncology patients (ages 18–27, median 23.5) enrolled in a fertility preservation program (diagnoses: Hodgkin’s lymphoma, Non-Hodgkin’s lymphoma, chronic myeloid leukemia). Informed consent was obtained from patients whose tissue was collected for this study. Three fragments from each patient were cryopreserved, thawed [21–25], and evaluated. The fragments in each group are 2–3 mm*3–4 mm*0.5–1.5 mm (3 to 18mm^3^). Four groups were formed:. Group 1: quick thawing of frozen ovarian tissue in boiling water (100 °C). Group 2: quick thawing of frozen ovarian tissue in boiling water (100 °C) and evaluated after 7 days of in vitro 3D culture. Group 3: slow thawing of frozen ovarian tissue in 37 °C water. Group 4: slow thawing of frozen ovarian tissue in 37 °C warm water and evaluated after 7 days of in vitro 3D culture (Fig. 1).

### Dynamic 3D in vitro culture with TISSEEL fibrin sealant

TISSEEL fibrin sealant (Baxter International Inc., Deerfield, IL, USA) was used to encapsulate ovarian tissue for subsequent in vitro culture and analysis. The fibrin sealant contains two main components: thrombin and fibrinogen, with final concentrations of 10 IU/mL and 45.5 mg/mL, respectively, to achieve optimal gel formation and encapsulation. The two components were quickly mixed in an Eppendorf tube using a pipette and vortexed. Then, 100 µL of the solution was dropped onto the ovarian tissue to form a nearly gel-like mixture. The gelation time is nearly 10 s. And the temperature is room temperature. After gelation was complete, the formed fibrin gel was gently peeled off using sterile forceps to avoid any damage to the tissue during in vitro culture. The encapsulated tissue was then immediately transferred to a 700 mL cell culture flask (Greiner Bio-One GmbH, Frickenhausen, Germany) containing 70 mL of culture medium. Seven-day cultures of ovarian tissue were performed in α-modified Eagle’s minimum essential medium (α-MEM, Life Technologies, Carlsbad, CA, USA) supplemented with 15% fetal bovine serum, 0.1 mg/mL streptomycin, and 100 IU/mL penicillin. The culture was placed in a humidified incubator at 37 °C with 5% CO_2_ and agitated at 75 times per minute using a rotary shaker. Half of the culture media was replaced every 48 h.

### Histological analysis

Ovarian tissues were fixed in 3.5% paraformaldehyde for 24 h at 4 °C and then embedded in paraffin. Four µm thick sections were cut, and every 10th section was mounted on glass slides and stained with hematoxylin and eosin. Morphological analysis of tissue development and viability was performed under a Nikon Diaphot 300 microscope at 200x and 400x magnification.

### Sequencing and data extraction

RNA extraction was performed for each ovarian tissue sample using the Trizol method [26, 27]. RNA integrity was assessed using the Bioanalyzer 2100 system (Agilent Technologies, CA, USA). Messenger RNA was purified from total RNA using poly-T oligonucleotide-linked magnetic beads. After fragmentation, first-strand cDNA was synthesized using random hexamer primers. Second-strand cDNA was then synthesized using dUTP instead of dTTP. After end repair, A-tailing, adapter ligation, size selection, amplification, and purification, the directional library was ready. The library was checked for quantification using Qubit and real-time PCR, and size distribution was detected using a bioanalyzer. After library quality control, different libraries were pooled together based on effective concentration and target data volume, and then subjected to Illumina sequencing. The basic principle of sequencing is “sequencing by synthesis”, where fluorescently labeled dNTPs, DNA polymerase, and adapter primers are added to the sequencing flow cell for amplification. As each sequencing cluster extends its complementary chain, the addition of each fluorescently labeled dNTP releases a corresponding fluorescent signal. The sequencer captures these fluorescent signals and converts them into sequencing peaks by computer software to obtain the sequence information of the target fragments. The reference genome and gene model annotation files were downloaded directly from the genome website. The index of the reference genome was built using Hisat2 v2.0.5, and the paired-end clean reads were aligned to the reference genome using Hisat2 v2.0.5. We chose Hisat2 as the mapping tool because Hisat2 can generate a splice site database based on the gene model annotation file, so the mapping results are better than other non-splicing mapping tools. The mapped reads of each sample were assembled by StringTie (v1.3.3b). StringTie uses a novel network flow algorithm with an optional de novo assembly step to assemble and quantify full-length transcripts representing multiple splice variants for each gene locus.

### Differential expression analysis

For DESeq2 with biological replicates: Differential expression analysis of two conditions/groups was performed using the DESeq2 R package (1.20.0). DESeq2 provides statistical procedures to determine differential expression in digital gene expression data using a model based on the negative binomial distribution. The resulting P values ​​were adjusted using the method of Benjamini and Hochberg to control the false discovery rate. Corrected P values ​​≤ 0.05 and |log2(foldchange)| ≥ 1 were set as thresholds for significant differential expression.

### KEGG enrichment analysis of differentially expressed genes

KEGG is a database resource for understanding the high-level functions and utility of biological systems (e.g., cells, organisms, and ecosystems) from molecular-level information, especially large-scale molecular datasets generated by genome sequencing and other high-throughput experimental technologies (http://www.genome.jp/kegg/). We used the R package cluster profile to test the statistical enrichment of differentially expressed genes in KEGG pathways.

### Gene set enrichment analysis

Gene set enrichment analysis (GSEA) is a computational method used to determine whether predefined gene sets can show significant differences between two biological states. Genes are ranked according to the degree of differential expression in the two samples, and then predefined gene sets are tested to see if they are enriched at the top or bottom of the list. Gene set enrichment analysis can include subtle expression changes. We used a local version of the GSEA analysis tool http://www.broadinstitute.org/gsea/index.jsp, and the KEGG datasets were used for GSEA separately.

## Results

### Morphology

**Fig. 2:**
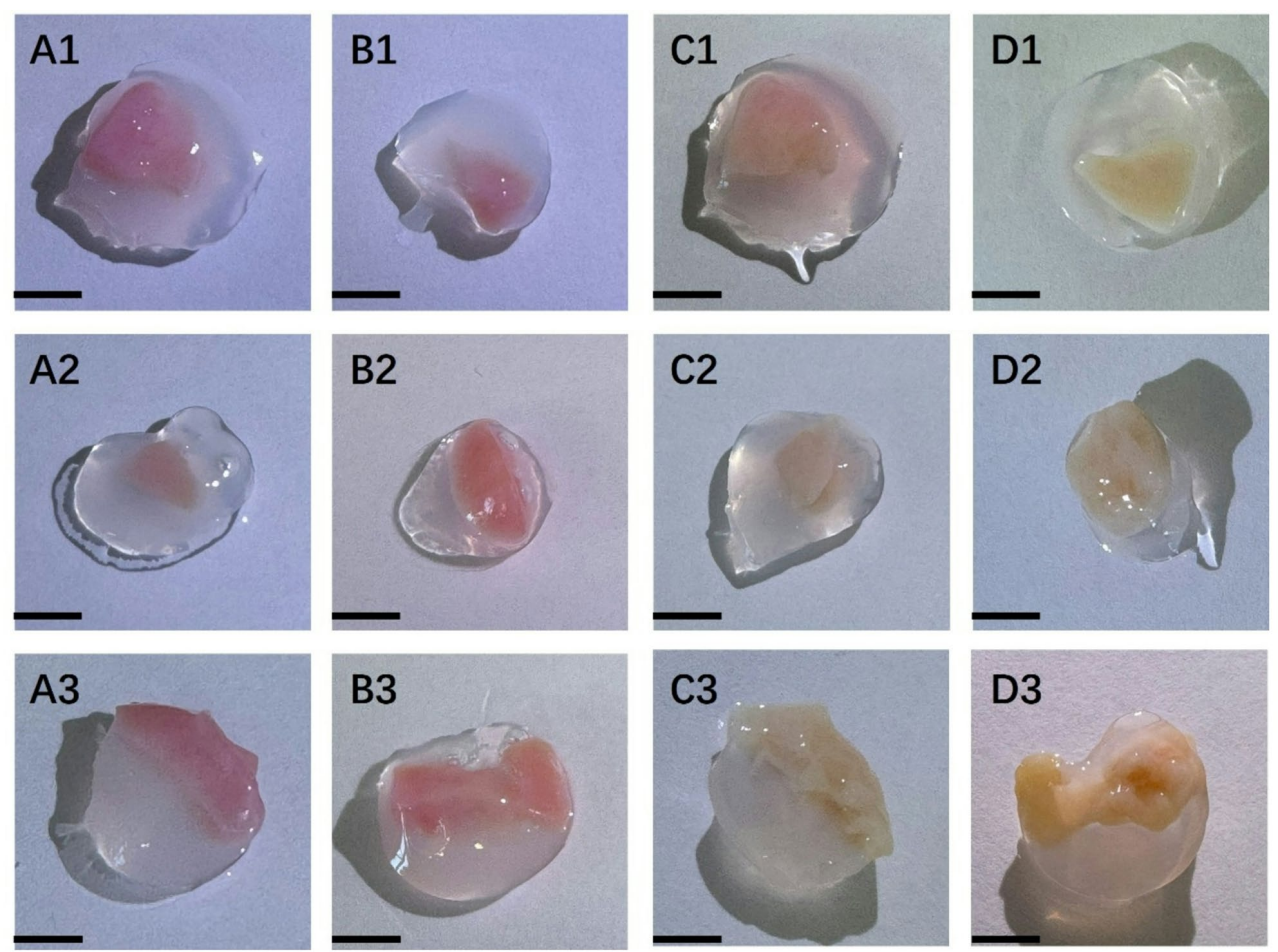
Cryopreserved ovarian tissue in vitro 3D culture with TISSEEL Fibrin. (A1–A3) Quick thawing cryopreserved ovarian tissue encapsulated in TISSEEL Fibrin sealant. (B1-B3) Slow thawed tissue encapsulated in TISSEEL Fibrin sealant. (C1-C3) Quick thawed tissue encapsulated in TISSEEL Fibrin sealant and cultured for 7 days. (D1-D3) Slow thawed tissue encapsulated in TISSEEL Fibrin sealant and cultured for 7 days. Bar = 2.5 mm

Ovarian tissue after quick thawing was well encapsulated in TISSEEL Fibrin Sealant, displaying a 3D full-surround effect (Fig. 2A1, 2A2, and 2A3). It was detected similarly well-wrapped the slow thawing ovarian tissue with TISSEEL Fibrin Sealant (Fig. 2B, 2B2, and 2B2). In Fig. 2C1, 2C2, and 2C3, after quick thawing and 7 days in vitro culture, the ovarian tissue turns light yellow. It may be that the enhanced metabolism of the tissue further leads to cell adaptation. Figure 2D1, 2D2, and 2D3 also show a similar color change of tissues after slow thawing and culture.

.

**Fig. 3:**
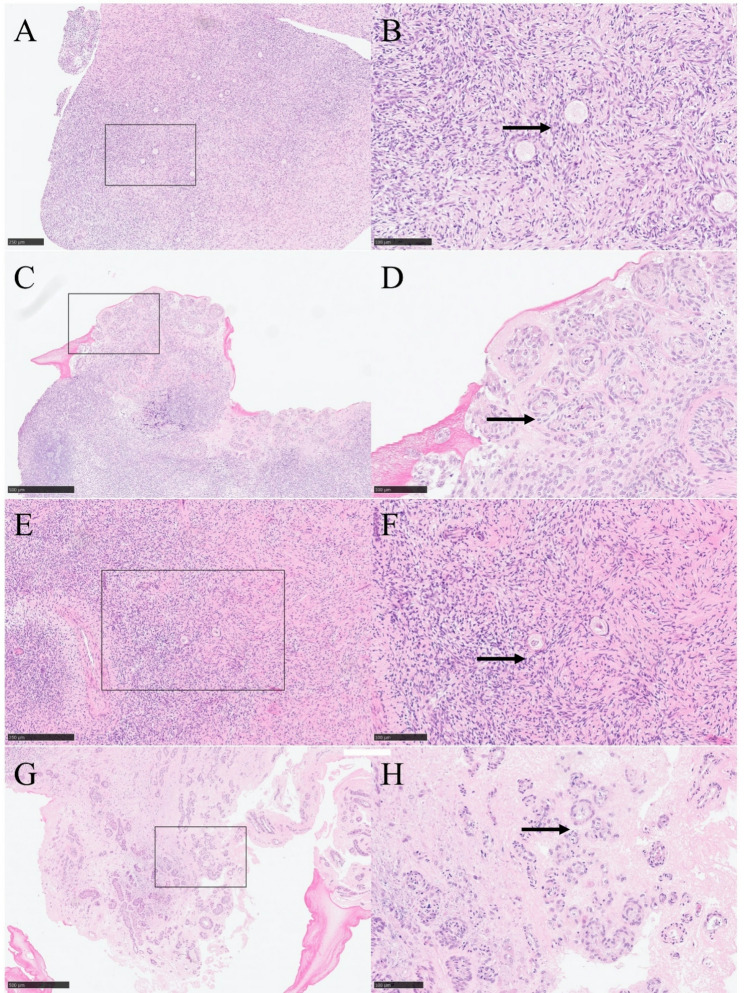
Hematoxylin-Eosin (HE)-staining of cryopreserved and in vitro cultured ovarian tissue. **A**, **B** Group 1 (quick thawed). **C**, **D** Group 2 (quick thawed and then in vitro 3D cultured). **E**, **F** HE-staining of Group 3 (slow thawed). **G**, **H** Group 4 (slow thawed and then in vitro 3D cultured). Bar for (**A**, **E**) = 250 μm, Bar for (**C**, **G**) = 500 μm, Bar for (**B**, **D**, **F**, **H**) = 100 μm.Arrows point to follicles

Figure 3A and B display hematoxylin-eosin (HE) staining of ovarian tissues that underwent quick thawing without following in vitro culture. Figure 3C and D present HE-stained sections of ovarian tissues subjected to quick thawing followed by a seven-day in vitro 3D culture. Notably, the follicle cells showed a tendency to develop and actively migrate into the fiber. Before cryopreservation, the ovarian medulla was partially removed to minimize the formation of intracellular ice crystals. Therefore, these sections have not shown mature follicles or secondary oocytes, only limited primordial follicles, which are the basis for future ovarian tissue development. The similar results are shown in Fig. 3E, F and G, and 3H. Compared to those observed in Fig. 3E and F of slow-thawed tissue that was not cultured, Fig. 3G and H illustrate the results of slow-thawed cryopreserved ovarian tissue after culture. Follicle cells showed a tendency to develop and actively migrate into the fiber. These results may suggest that in vitro 3D culture can effectively promote the growth, development, and migration of follicle cells.

### Differential Expression Genes (DEGs)

**Fig. 4:**
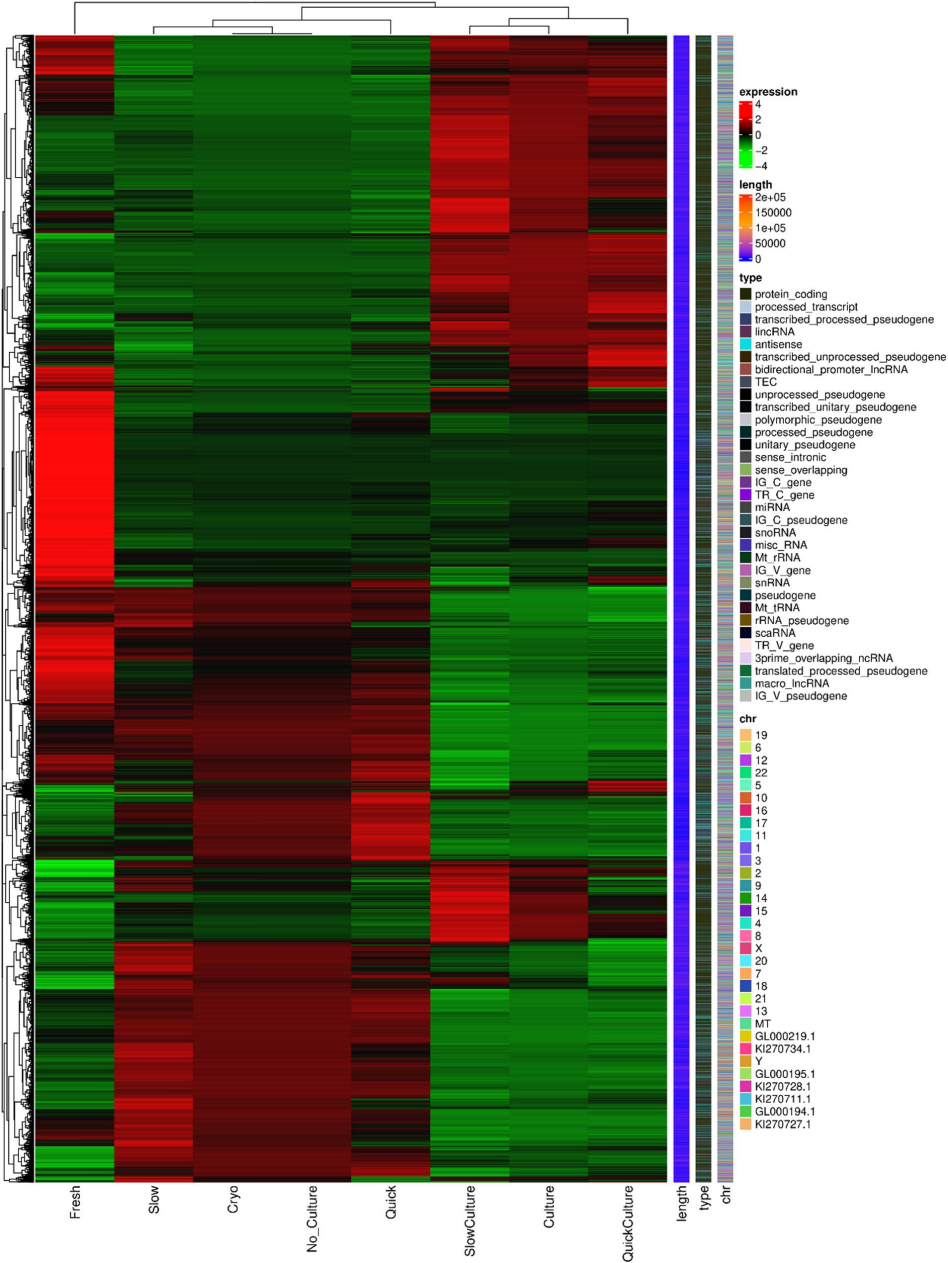
Heatmap of differential expressed genes (DEGs)

**Fig. 5:**
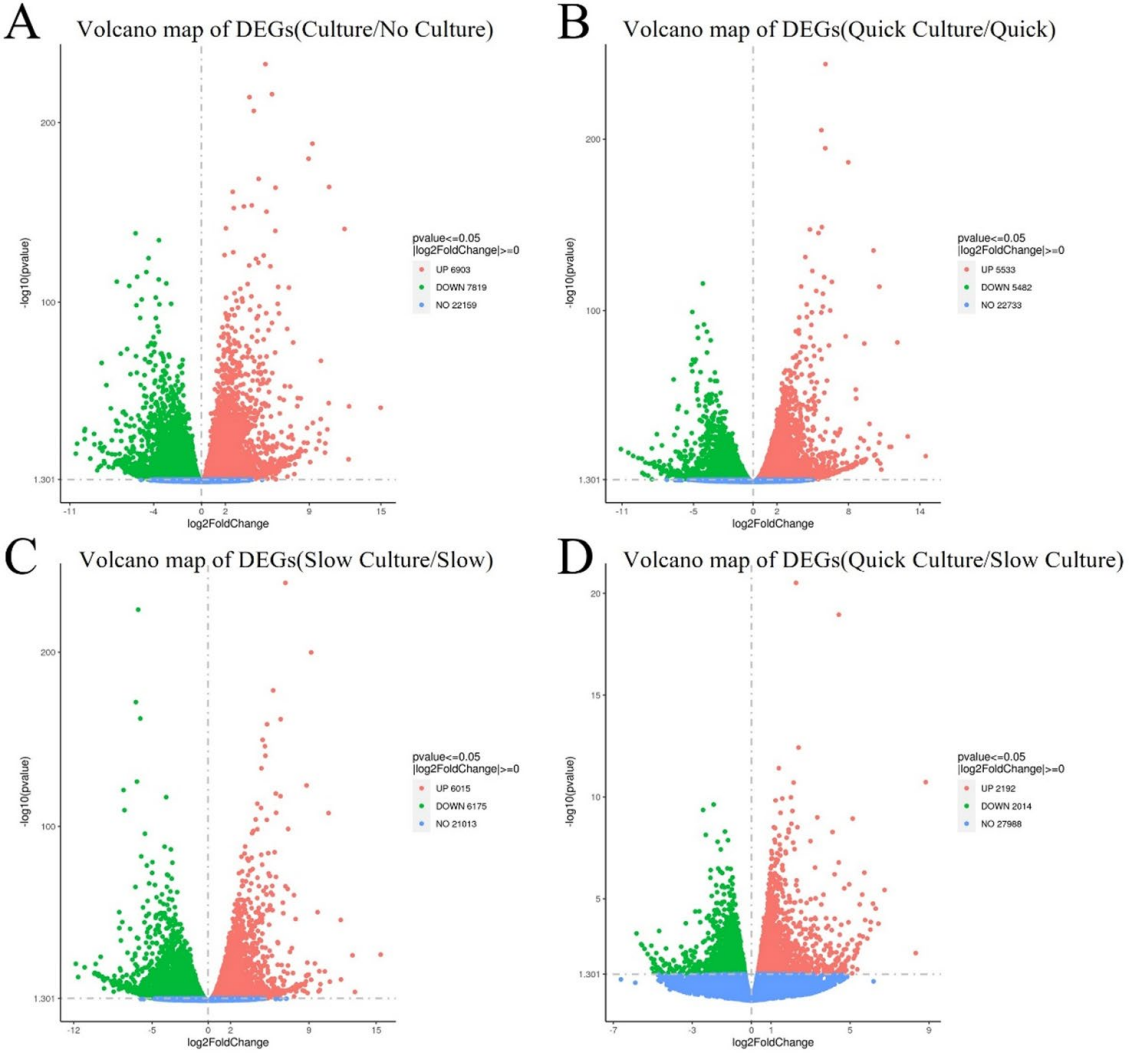
Volcano map of differential expressed genes (DEGs). **A** DEGs volcano map: Groups 2 and 4 (thawing and then in vitro 3D culture) vs. Groups 1 and 3 (thawing without culture). **B** DEGs volcano map: Group 2 (quick thawing ovarian with culture) vs. Group 1 (quick thawing). **C** DEGs volcano map: Group 4 (slow thawing ovarian tissue with culture) vs. Group 3 (slow thawing). **D** Group 2 (quick thawing with culture) vs. Group 4 (slow thawing with culture).

The heatmap of DEGs is shown in Fig. 4. Volcano plots were generated to compare the up-regulated and down-regulated differentially expressed genes in the tissues of different groups. Comparison of cells in Group 1 and Group 3 (thawing of ovarian tissue without culture), 6903 genes were up-regulated and 7819 genes were down-regulated in groups 2 and 4 (thawing with in vitro 3D culture), as shown in Fig. 5A. Compared with Group 1 (quick thawing), 5533 genes were up-regulated and 5482 genes were down-regulated in cells of Group 2 (quick thawing with in vitro 3D culture), as shown in Fig. 5B. Meanwhile, compared with cells of Group 3 (slow thawing), 6015 genes were up-regulated and 6175 genes were down-regulated in cells of Group 4 (slow thawing with in vitro 3D culture), as shown in Fig. 5C. Compared with cells of Group 2 (quick thawing with in vitro 3D culture), 2192 genes were up-regulated and 2014 genes were down-regulated in cells of Group 4 (slow thawing with in vitro 3D culture), as shown in Fig. 5D.

### Kyoto Encyclopedia of Genes and Genomes (KEGG)

**Fig. 6:**
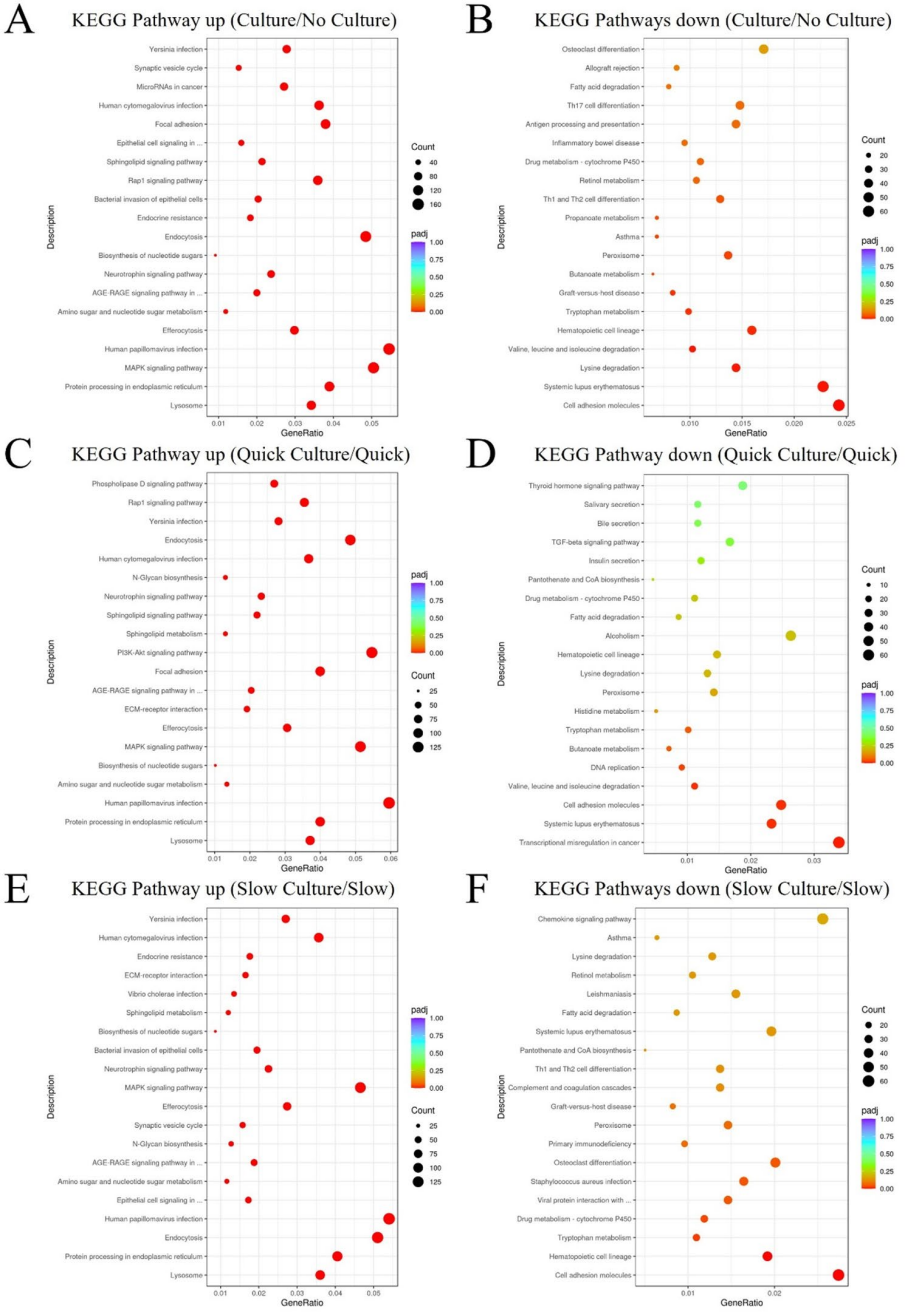
Visualization dot map of Kyoto Encyclopedia of Genes and Genomes (KEGG) pathway analysis. **A** KEGG pathway analysis up-related: cells of Group 2 and Group 4 (thawing of ovarian tissue with in vitro 3D culture) vs. cells of Group 1 and Group 3 (thawing without culture). **B** KEGG pathway analysis down-related: cells of Group 2 and Group 4 (thawing of ovarian tissue with culture) vs. cells of Group 1 and Group 3 (thawing of ovarian tissue without culture). **C** KEGG pathway analysis up-related: Group 2 (quick thawing of ovarian tissue with culture) vs. Group 1 (quick thawing). **D** KEGG pathway analysis down-related: Group 2 (quick thawing with culture) vs. Group 1 (quick thawing). **E** KEGG pathway analysis up-related: Group 4 (slow thawing ovarian tissue with culture) vs. Group 3 (slow thawing). **F** KEGG pathway analysis down-related: Group 4 (slow thawing with culture) vs. Group 3 (slow thawing)

KEGG pathway enrichment analysis based on DEGs. In comparison to Group 1 and Group 3 (thawing of ovarian tissue without culture), DEGs in cells of Group 2 and Group 4 (thawing of ovarian tissue with in vitro 3D culture), are mainly enriched and up-related in the lysosome pathway and protein processing in the endoplasmic reticulum pathway (Fig. 6A) and mainly down-related in the cell adhesion molecules pathway (Fig. 6B). Compared to cells of Group 1(quick thawing), DEGs in cells of Group 2 (quick thawing with culture) are also mainly enriched and up-related in the lysosome pathway and protein processing in the endoplasmic reticulum pathway (Fig. 6C) and mainly down-related in the cell adhesion molecules pathway (Fig. 6D). Compared to cells of Group 3 (slow thawing), DEGs in cells of Group 4 (slow thawing with culture) are also mainly enriched and up-related in the lysosome pathway and protein processing in the endoplasmic reticulum pathway (Fig. 6E) and mainly down-related in the cell adhesion molecules pathway (Fig. 6F).

### Gene Set Enrichment Analysis (GSEA)

**Fig. 7:**
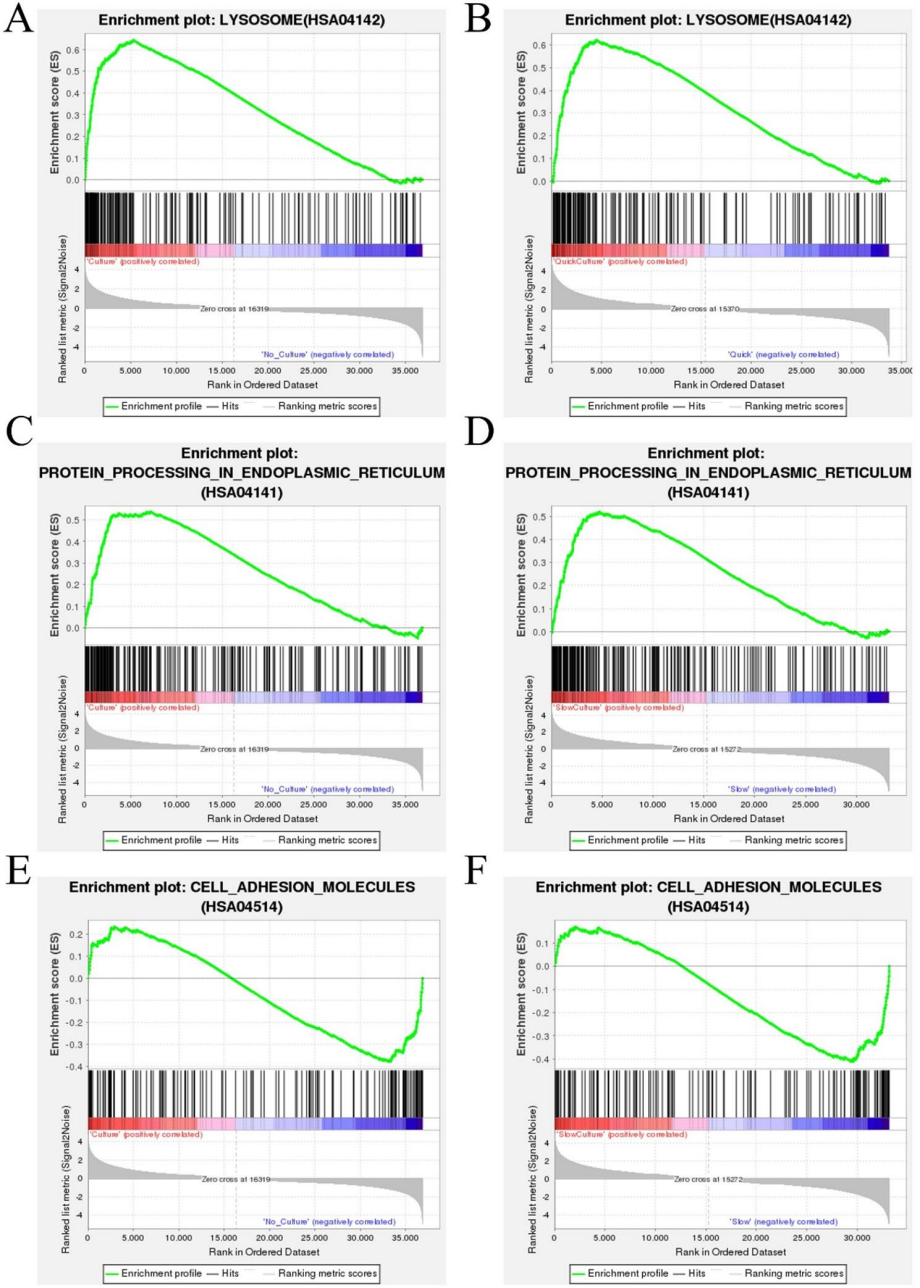
Gene Set Enrichment Analysis (GSEA) based on KEGG pathway data. **A** GSEA analysis indicated the lysosome pathway was enriched and up-regulated in cells of groups 2 and 4 (thawing of ovarian tissue with in vitro 3D culture) vs. cells of groups 1 and 3 (thawing without culture). **B** GSEA analysis indicated the lysosome pathway was enriched and up-regulated in cells of Group 2 (quick thawing with culture) vs. cells of Group 1 (quick thawing). **C** GSEA analysis indicated the protein processing in the endoplasmic reticulum pathway was enriched and up-regulated in cells of groups 2 and 4 (thawing ovarian tissue with culture) vs. cells of groups 1 and 3 (thawing without culture). **D** GSEA analysis indicated the protein processing in the endoplasmic reticulum pathway was enriched and up-regulated in cells of Group 4 (slow thawing with in vitro 3D culture) vs. cells of Group 3 (slow thawing). **E** GSEA analysis indicated the cell adhesion molecules pathway was enriched and down-regulated in cells of groups 2 and 4 (thawing with culture) vs. cells of groups 1 and 3 (thawing without culture). **F** GSEA analysis indicated the cell adhesion molecules pathway was enriched and down-regulated in cells of Group 4 (slow thawing with culture) vs. cells of Group 3 (slow thawing)

GSEA is an in-depth data analysis based on predefined gene sets in the KEGG database. In comparison to cells of groups 1 and 3 (thawing of ovarian tissue without in vitro 3D culture culture), enrichment of genes involved in the lysosome pathway in upregulation in cells of groups 2 and 4 (thawing with culture) (Fig. 7A). The enrichment of genes involved in the lysosome pathway in upregulation also in cells of Group 2 (quick thawing with culture) compared to cells of Group 1(quick thawing) (Fig. 7B). It is shown that the expression of the lysosomal pathway in thawed ovarian tissue followed in vitro 3D culture was significantly upregulated compared to that after thawing without culture.

Meanwhile, compared to groups 1 and 3 (thawing without culture), enrichment of genes involved in the protein processing in the endoplasmic reticulum pathway in upregulation in cells of groups 2 and 4 (thawing with culture) (Fig. 7C). The enrichment of genes involved in the protein processing in the endoplasmic reticulum pathway in upregulation is also in cells of Group 4 (slow thawing with culture) compared to cells of Group 3 (slow thawing) (Fig. 7D). It is shown that the expression of the protein processing in the endoplasmic reticulum pathway in thawing ovarian tissue with in vitro 3D culture was significantly upregulated compared to that in thawing ovarian tissue without culture.

Compared to cells of groups 1 and 3 (thawing without culture), cells of groups 2 and 4 (thawing with culture) shown downregulation of genes involved in the cell adhesion molecules pathway (Fig. 7E), and compared to cells of Group 4 (slow thawing with culture), cells of Group 3 (slow thawing) also shown downregulation of genes involved in the cell adhesion molecules pathway (Fig. 7F). It is noted that the expression of the cell adhesion molecules pathway in thawing ovarian tissue with in vitro 3D culture was significantly downregulated compared to that in thawing ovarian tissue without culture.

## Discussion

### Effects of in vitro 3D culture on follicle growth in ovarian tissue

The primary goal of in vitro culture after ovarian tissue cryopreservation (freezing and thawing) is to preserve ovarian tissue function and enhance follicle growth, which is essential for successful transplantation. However, the development of follicles is critical in vitro culture [28, 29].

In the natural ovarian microenvironment, inhibitory factors such as anti-Müllerian hormone (AMH) maintain primordial follicles in a dormant state. However, in vitro culture may weaken these inhibitory signals, leading to premature follicle activation [30, 31]. Additionally, high-glucose culture media accelerates ovarian tissue metabolism, further promoting follicular development.

3D culture systems provide a spatially supportive environment for follicular growth, facilitating interactions between granulosa cells and oocytes while preserving the microenvironment necessary for normal development. TISSEEL Fibrin, used in our study, mimics the ovarian extracellular matrix, offering a stable 3D scaffold that maintains follicular architecture and enhances follicle survival rates.

Results of our experiments demonstrated that in vitro 3D culture with TISSEEL Fibrin may promote follicular growth in ovarian tissue. As shown in Fig. 3, after 3D in vitro culture, the follicles have developed a tendency. Notably, in certain areas, a follicle colony phenomenon, which is multifollicular development, is observed. It can be characterized by the simultaneous development of multiple follicles close.

While follicular growth and development are beneficial, excessive follicle activation and clustering may accelerate follicle depletion. The early transplantation of dominant follicles or their selection for IVF could be advantageous. However, for long-term follicular viability after transplantation of ovarian tissue, minimizing excessive follicle activation during in vitro culture is critical [32].

The formation of follicle colonies may lead to competition for nutrients, potentially causing metabolic insufficiency, impaired growth, and apoptosis at later stages. The addition of localized inhibitory factors, such as AMH, during culture may help regulate follicular activation, mitigating excessive simultaneous development while supporting controlled follicle maturation [31].

As shown in the KEGG pathway (Fig. 5), both quick and slow thawing methods resulted in significant upregulation of the endoplasmic reticulum (ER) protein synthesis pathway in ovarian tissues compared to the tissue from uncultured groups. This finding was further supported by the GSEA analysis (Fig. 6), which confirmed the significant activation of ER protein synthesis. The ER protein synthesis pathway can promote signal recognition, translocation, folding, modification, and quality control of newly synthesized proteins, and plays a key role in follicular development and hormone receptor synthesis. When external conditions are changing, the ER may undergo a stress response to enhance cell adaptability. Under ER stress conditions, cells with excessive misfolded proteins undergo apoptosis, while others activate adaptive mechanisms to enhance survival [33–35]. The upregulation of ER protein synthesis suggests heightened cellular metabolism and active tissue growth, aligning with the observed morphological adaptations.

### Effects of in vitro 3D culture on cell adhesion

The main advantage of in vitro 3D culture over direct culture is that it provides a spatially structured environment that preserves natural adhesion between cells, which is essential for follicular development [36, 37]. Cell adhesion molecules, especially cadherins, play a vital role in mediating cell-cell adhesion, maintaining tissue structure, and regulating cell function [38]. In ovarian tissue culture, cadherins promote interactions between oocytes and granulosa cells, thereby preserving follicular structure and function [39]. Therefore, ensuring that the 3D scaffold structure is stable and maintaining cell adhesion is essential to support follicle growth.

In addition, TISSEEL Fibrin in 3D culture can act as a seamless tissue adhesive, providing potential support for applications beyond in vitro culture [40]. During ovarian transplantation, minimizing mechanical damage is essential for the successful implantation of tissues. Whether the fibrin-based 3D culture system can be directly transplanted into patients, thereby reducing the need for suturing, limiting mechanical trauma, and promoting seamless integration of ovarian tissue with the host environment is what we want to achieve.

Our study found that in vitro 3D culture with TISSEEL Fibrin weakened cell adhesion in ovarian tissue. HE-stained sections of uncultured ovarian tissue in the control group showed tight cell junctions (Fig. 3). However, after 7 days of in vitro 3D culture with TISSEEL Fibrin, cell junctions were significantly weakened, and intercellular gaps became more obvious.

Our in vitro 3D culture is designed to preserve the spatial structure of ovarian tissue. Too tight adhesion in uncultured tissue may hinder the growth of follicles, but excessive relaxation of cell junctions observed after culture may impair nutrient transport, which is essential for the development of follicles. Tissue relaxation may result from prolonged culture duration, inadequate nutrient supply, or an imbalance in the proliferation rates of follicles and surrounding stromal cells. Additionally, a slight reduction in fibrin was observed after culture, indicating its gradual degradation during the culture period. This fibrin loss may contribute to the collapse of the 3D scaffold, leading to structural instability, disruption of the intercellular space, and weakening of cell adhesion.

In addition, follicular cells showed a tendency to grow into fibrin colloids, indicating potential follicular migration. Some follicles developed in residual colloids, suggesting that, in addition to providing structural support, fibrin-based 3D scaffolds can promote follicle migration (Fig. 3B). If such scaffolds are applied in vivo, they may accelerate the integration of transplanted ovarian tissue into the host environment.

As shown in the KEGG pathway analysis (Fig. 5), the cell adhesion molecule pathway was significantly downregulated in cultured ovarian tissue compared to uncultured tissue, regardless of whether a quick or slow thawing method was applied. The GSEA analysis (Fig. 6) further confirmed this downregulation. A reduction in cell adhesion molecules weakens cell-cell and cell-matrix interactions, potentially compromising cellular morphology and tissue integrity, and, in severe cases, leading to cell disintegration [41]. This decline in adhesion molecules may be associated with alterations in the extracellular matrix composition. As culture time increases, follicular cell proliferation and ovarian microenvironmental changes may further impact the 3D culture system. Optimizing culture duration could mitigate these effects. Additionally, increased cellular metabolism within the tissue may result in nutrient depletion, inducing cellular stress and further reducing the expression of cell adhesion molecules. The addition of appropriate cell adhesion molecules to the culture medium may enhance its structural stability.

### Effects of in vitro 3D culture on cell damage

During the in vitro culture of ovarian tissue, cell damage is a common issue. Irreversible damage, caused by factors such as hypoxia, malnutrition, accumulation of metabolic waste, or mechanical damage, can further induce cell autophagy, eventually leading to cell necrosis [42–44].

In our study, it was observed that prior to culture, the ovarian tissue appeared light pink, indicating a rich internal blood supply and healthy tissue condition (Fig. 2). After 7 days of culture, the tissue color changed to light yellow, suggesting that the internal blood supply was compromised, potentially due to ischemia and hypoxia. HE-stained sections revealed significant follicular cell proliferation and the formation of follicular colonies, which indicated an increase in internal tissue metabolism (Fig. 3). However, this metabolic increase likely resulted in nutrient depletion and inefficient metabolic waste removal. Concurrently, the tissue gaps widened, cell connections weakened, and signs of apoptosis and necrosis were evident.

Several factors may contribute to this situation. First, the absence of follicle activation inhibitors and the optimal growth environment led to the simultaneous activation and development of multiple follicles, which generated an increased demand for nutrients and the production of metabolic waste. The ovarian tissue may have been unable to adapt to this heightened metabolic activity, resulting in cellular apoptosis. Second, the culture time may have been too long.

In our experiments, ovarian tissue was cultured in the medium for 7 days, and although the 3D scaffold mimics the in vivo environment, it does not offer the same precise regulation. Prolonged culture leads to insufficient tissue nutrition, metabolic waste accumulation, and consequent cell apoptosis. Furthermore, the slight reduction in fibrin after culture suggests fibrin loss during the culture period, which may contribute to the collapse of the 3D scaffold, disruption of the intercellular space structure, weakening of cell adhesion, and a decrease in cell supply, further leading to cell apoptosis.

The KEGG pathway analysis (Fig. 5) reveals that, regardless of whether the tissue underwent rapid or slow recovery, the lysosomal pathway in the cultured ovarian tissue was significantly upregulated compared to uncultured tissue. Further confirmation of this upregulation is provided by the GESA analysis (Fig. 6), which underscores the increased activity of the lysosomal pathway in the cultured ovarian tissue. The upregulation of the lysosomal pathway indicates enhanced cellular autophagy. Autophagy is a process by which cells adapt to stress and manage their metabolic needs. Damaged organelles, proteins, and other cellular debris are encapsulated into autophagosomes and subsequently degraded by lysosomes [45–48]. This increase in autophagic activity suggests that during the culture, cell metabolism was heightened, triggering cellular stress and activating autophagy mechanisms.

According to the analysis presented above, in the future, an appropriate amount of AMH can be added to inhibit the simultaneous activation of follicles, ensuring the growth and development of a certain number of follicles, and reducing metabolic activities in the tissue. It can help reduce a reduction of time of culture to ensure that the tissue is transplanted in a good state and avoid cell stress caused by insufficient nutrient supply. The volume of the 3D scaffold can also be further expanded to ensure the 3D support function during the entire culture process and the natural adhesion state between cells.

Luyckx V et al. ’s study used different concentrations of human fibrinogen and thrombin to systematically test the mechanical strength, degradation rate, pore structure, and cell compatibility of the resulting gels. In the experiments, they embedded human ovarian stromal cells in these fibrin scaffolds and observed their cell adhesion, diffusion, growth, and survival during in vitro culture. The results showed that appropriate concentration ratios (especially moderate concentrations of fibrinogen and thrombin) can form stable and biodegradable three-dimensional scaffolds that support good survival and morphological maintenance of ovarian stromal cells [49].

Their team also first isolated primordial and primary follicles from mouse ovaries and then co-embedded these follicles with ovarian stromal cells in fibrin gels of varying ratios. Subsequently, the complex was autologously transplanted back into the mice (subcutaneously or around the ovary), and samples were taken several weeks later to observe follicle survival, proliferation, and structural integrity. The results showed that the fibrin scaffold effectively maintained the three-dimensional structure of the follicles, promoted the interaction between the follicles and surrounding cells, and supported the continued growth and angiogenesis of the follicles after transplantation [50]. These findings are consistent with similar findings reported in this paper, namely that similar fibrin three-dimensional scaffolds may promote follicle growth.

Wang et al. have also used the TISSEEL Fibrin. They embedded primordial follicles isolated from human ovarian tissue into a fibrin scaffold for in vitro culture. They then used confocal microscopy and three-dimensional imaging technology to automatically assess the morphology, activity, and development of the follicles. The results showed that some follicles were successfully activated and continued to develop during culture, while others died [19]. Their research only applied it to follicles, while we applied it to ovarian tissue, further validating the advantages of this three-dimensional culture method, but also further exposing its problems. We need to continuously research and optimize this technology.

### Limitations

This study examined two thawing methods for ovarian tissue cultured in a TISSEEL Fibrin 3D system and analyzed transcriptomic differences via RNA sequencing. In vitro 3D-cultivation of ovarian tissue for 7 days with TISSEEL Fibrin is may promote follicular growth, actively migrate into the fiber, weaken cell adhesion, and cause little cell damage, though validation in larger studies is needed due to limited sample size.

## Conclusions

The technology of the described dynamic in vitro 3D-cultivation of ovarian tissue for 7 days with TISSEEL Fibrin is informative and demonstrates that it may promote follicular growth, actively migrate into the fiber, weaken cell adhesion, and cause little cell damage.

## Data Availability

The raw data of RNA-seq can be downloaded at “Sequence read archive” on the National Center for Biotechnology Information (https://dataview.ncbi.nlm.nih.gov/object/PRJNA1188025?reviewer=or17fgchi8hki9n00bv4e3n1b3, accessed on 12 December 2024).
